# Nbeal2 Inactivation Triggers Abl1 Stabilisation and Dysregulated Subcellular Localisation of the Multi‐Drug‐Resistant Protein MDR1 (ABCB1) in Mast Cells

**DOI:** 10.1111/imm.70055

**Published:** 2025-10-19

**Authors:** Raphaela Marquardt, Nico Andreas, Ronja Herrnberger, Claudia Küchler, Katrin Hornung, Bernhard Nieswandt, Marco Groth, Paul M. Jordan, Oliver Werz, Julia Drube, Sebastian Drube

**Affiliations:** ^1^ Institut für Immunologie Universitätsklinikum Jena, Friedrich‐Schiller‐Universität Jena Jena Germany; ^2^ Institute of Experimental Biomedicine University Hospital Würzburg and Rudolf Virchow Center for Integrative and Translational Bioimaging Würzburg Germany; ^3^ CF Next‐Generation Sequencing Fritz Lipmann Institute Jena Germany; ^4^ Department of Pharmaceutical/Medicinal Chemistry, Institute of Pharmacy Friedrich Schiller University Jena Jena Germany; ^5^ Institut für Molekulare Zellbiologie Universitätsklinikum Jena, Friedrich‐Schiller‐Universität Jena Jena Germany

**Keywords:** ABCB1, Abl1, mast cell, Nbeal2

## Abstract

Inactivation of the BEACH (*beige and chediak‐higashi*) family member NBEAL2 in humans and mice results in the development of the gray platelet syndrome (GPS), a bleeding disorder characterised by macrothrombocytopenia and splenomegaly. On the cellular level, NBEAL2 inactivation leads to functional defects in megakaryocytes, platelets, neutrophils and NK cells. In addition, *Nbeal2* deletion in mice causes specific defects in mast cells (MCs), such as accumulation of transcription factors and proteins that are involved in the protein biosynthesis machinery. These defects culminate in non‐physiological survival behaviour and an amplified stem cell factor (SCF)‐, interleukin (IL)‐3‐ and IL‐33‐induced cytokine production. Here we show that NBEAL2 deficiency also leads to an Abl1‐supported increased surface expression of the multi‐drug‐resistant protein 1 (Mdr‐1) ABCB1, which is antagonised by the IL‐33‐induced activation of the TAK1‐IKK2 module and the activation of Src‐family‐kinases (SFKs). Our data demonstrate that NBEAL2 is required to control the surface expression of ABCB1.

## Graphical abstract

(A) Proposed model for Abl1 degradation via Nbeal2. Nbeal2 interacts with Abl1 with its WDR domain. This results in proteasomal degradation by a yet unidentified ubiquitin ligase. Therefore, *Nbeal2* inactivation stabilises Abl1, which supports mast cell (MC) degeneration. (B) In wt MCs, the Abl1 protein level is controlled by Nbeal2. Furthermore, Nbeal2 promotes the generation and stability of ABCB1‐loaded MC granules within wt MCs. In *Nbeal2* ^−/−^ MCs the stability of ABCB1‐loaded granules is decreased and the translocation of ABCB1 to the cell membrane is supported by stabilised Abl1.

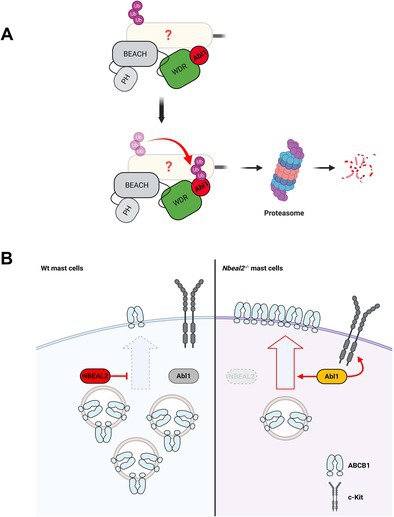

## 1 Materials and Methods

### 1.1 Mice

We used sex‐ and age‐matched (8–12 weeks) wt and *Nbeal2* ^−/−^ mice (littermates). Mice were maintained at the Animal Research Facility of the Jena University Hospital. Isolation of organs and cells was approved by the Thüringer Landesamt für Lebensmittelsicherheit und Verbraucherschutz (TLLV); Bad Langensalza; licence for the Institute of Immunology, Jena is twz‐36‐2017 and twz‐08‐2023.

### 1.2 Generation of Bone Marrow Derived Mast Cells (BMMCs)

Bone marrow cells from wt and *Nbeal2* ^−/−^ mice were cultured in IMDM (Gibco) supplemented with 10% FCS, 100 U/mL penicillin, 100 mg/mL streptomycin and 50 mM 2‐mercaptoethanol (complete medium) as well as supplemented with IL‐3‐containing (20 ng/mL) conditioned medium from X63Ag‐653 BPV‐rmIL‐3 cells. Adherent cells were removed from the cultures by refreshing the media and the culture bottles every second day during the generation of BMMCs for 4 weeks. In some experiments, during the BMMC generation [in absence or presence of IL‐33 (50ng/mL, Peprotech)] cultures were tested for the expression of the multi‐drug resistant protein1 (ABCB1) on c‐Kit+ cells by flow cytometry. To determine the maturation of mast cells, the content of CD117+/FcεRI+ MCs (independently from the expression of ABCB1) must have reached 95% after 4 weeks in culture. In these cases, the cultures were used for experiments for additional 4–5 weeks with refreshing the media twice a week.

### 1.3 Culturing of Cell Lines

P815, HMC‐1.1, HMC‐1.2, parental and *Nbeal2*KO MC/9 cells were cultured in RPMI (Gibco) supplemented with 10% FCS, 100 U/mL penicillin, 100 mg/mL streptomycin and 50 mM 2‐mercaptoethanol. For the culture of parental and *Nbeal2*KO MC/9 cells conditioned medium from X63Ag‐653 BPV‐rmIL‐3 cells was added (with an IL‐3 content of 20 ng/mL).

### 1.4 Expansion of Connective‐Tissue MCs (CTMCs)

To isolate CTMCs the peritoneal cavities of wt and *Nbeal2* ^−/−^ mice were flushed with ice‐cold PBS. To expand CTMCs from these isolations, peritoneal cavity cells were cultured in complete IMDM (Gibco) supplemented with SCF and IL‐3 (both 10 ng/mL, Peprotech). The purity of CTMCs was determined by staining for c‐Kit+/FcεRI+ cells after 2–3 weeks. When CTMC cultures reached the purity of 90%–95% c‐Kit+/FcεRI+ cells, cultures were used for 1 week. Thereby, one biological replicate consists of CTMCs from 5 wt or 5 *Nbeal2* ^−/−^ mice.

### 1.5 Treatment and Stimulation of BMMCs and CTMCs

BMMCs (1 × 10^6^ cells/mL) were treated with vehicle (DMSO) or the IKK‐inhibitor VII (1 μM), 5Z‐Oxozeanol (5 μM), U0126 (10 μM), L‐Skepinone (5 μM), SP600125 (5 μM), LY294002 (5 μM), Elacridar (5 μM), Dynasore (10 μM) or Nilotinib (5 μM) (all Selleckchem) for 30 min or as indicated in the figures. BMMCs or CTMCs were left unstimulated or were stimulated with IL‐33 (50 ng/mL, Peprotech) as indicated in the figure legends. In some experiments BMMCs we starved for survival factors or were stimulated with IL‐3 (50 ng/mL; Peprotech). BMMCs or CTMCs were either lysed for western blotting, analysed by flow cytometry or supernatants were collected and analysed by ELISA as recently described [1].

### 1.6 Lipid Mediator (LM) Metabololipidomics

Wt and *Nbeal2* ^−/−^ BMMCs (1 × 10^6^ cells/mL) were stimulated with IL‐33 (50 ng/mL, Peprotech) for 24 h in presence of IL‐3 (50 ng/mL, Peprotech). The supernatants were analysed for LMs as recently described [2, 3, 4]. The LM profile was determined by the Acquity UPLC system (Waters) and a QTRAP 5500 mass spectrometer (ABSciex) which was equipped with a TurboV source and electrospray ionisation. The quantification was achieved by calibration curves for each LM [4]. The linear calibration curves were obtained for each LM and gave *r* ^2^ values of 0.998 or higher (for fatty acids 0.95 or higher).

### 1.7 RNA Sequencing and Analysis

Sequencing data discussed here was downloaded from NCBI's Gene Expression Omnibus (see section Data availability). Processing of reads and detection of differentially expressed genes was done as described by [1].

### 1.8 Cell Lysis and Western Blotting

We used a lysis buffer containing 20 mM HEPES, pH7.5; 10 mM EGTA; 40 mM β‐glycerophosphate; 2.5 mM MgCl_2_; 2 mM orthovanadate; 1 mM dithiothreitol; 20 μg/mL aprotinin; 20 μg/mL leupeptin and 1% Triton. After the determination of the protein content by using the BCA‐kit (Pierce) samples were boiled in Laemmli buffer. Samples were separated by 10% sodium dodecyl sulphate (SDS)‐Laemmli gels. Proteins were transferred onto nitrocellulose membranes (SERVA) by Western blotting. Afterwards, membranes were blocked with dry milk, washed (in TBS with 0.1% Tween) and incubated with antibodies detecting phosphorylated (indicated by “p”) proteins and total proteins. We used pIKK1/2 (#2078), IKK1/2 (#61294), pMEK1/2 (#9154), MEK1/2 (#4694), pERK1/2 (#9106), ERK1/2 (#9102), pp38 (#9215), p38 (#9212), pJNK1/2 (#9251), JNK1/2 (#9262), ABCB1 (#13978), tubulin (#3873), p‐tyrosine (Y) (#9411), pan‐pY‐416 SFK (#6943), Lyn (#2732), c‐Kit (#3074), Fyn (#4023), pAbl1 (#2861), Abl1 (#2862) (all from Cell Signalling Technology), NBEAL2 (ab187162) (Abcam), IL‐33R (BAF1004) (R&D Systems) and Hck (sc‐1428) (Santa Cruz biotechnology). Membranes were washed in TBS with 0.1% Tween and were incubated with the HRP‐conjugated anti‐rabbit‐Ig (#5220–0336), or anti‐mouse‐Ig (#5220–0341) (both Medac GmbH). The ECL reagent (Pierce) was used for detection of the proteins.

### 1.9 Rhodamine123 (Rh.123) Assays for Wt and *Nbeal2* ^−/−^ BMMCs

Rh.123 is a substrate for ABCB1. Control samples (0 min) BMMCs were treated with Elacridar (5 μM) to block the efflux of Rh.123 whereas the samples for the kinetics were left untreated. Afterwards all samples were loaded with Rh.123 (1 μg/mL, Sigma‐Aldrich) for 30 min on ice. Then, BMMCs were transferred to 37°C and after the indicated time points, cells were harvested, samples were treated with Elacridar (to avoid further Rh.123 efflux), were washed with PBA (PBS with 5% BSA and 0.1% NaN_3_) and were stained for ABCB1.

### 1.10 Flow Cytometry

Cells (BMMCs, CTMCs, P815, HMC‐1.1 and HMC‐1.2) were harvested und washed with PBA. Unspecific bindings of antibodies were blocked by pre‐incubation with rat‐IgG (Jackson) at 4°C for 8 min. Subsequently, cells were stained with PE‐conjugated anti‐c‐Kit (#105808), FITC‐conjugated anti‐FcεRI (#134306), PE‐Cy7‐conjugated anti‐ABCB1 (#348610), PE‐Cy7‐conjugated anti‐IL‐33R (#146609) (all Biolegend). After 30 min, cells were washed with PBA buffer and prior to analysis, PI (Sigma) was added to determine the percentage of dead cells and to exclude them from further analyses. Samples were analysed with an LSR II flow cytometer (BD) and FlowJo V10.4.2 (BD).

### 1.11 Cell Sorting and Culturing of Sorted Cells

*Nbeal2* ^−/−^ BMMCs were stained with PE‐conjugated anti‐c‐Kit (#105808) and PE‐Cy7‐conjugated anti‐ABCB1 (#348610) (all Biolegend) for 30 min. BMMCs were sorted into ABCB1+ and ABCB1− BMMCs populations using the FACS Aria II (BD). Afterwards, ABCB1+ and ABCB1− *Nbeal2* ^−/−^ BMMCs were adjusted to a cell number of 1 × 10^6^ cells/mL and were cultured in IL‐3‐containing medium. After different time points ABCB1+ and ABCB1− BMMCs were harvested and stained as written in the flow cytometry section.

### 1.12 Western Blot Quantification

All Western blot experiments were performed with at least 3 biological replicates of wt or *Nbeal2* ^−/−^ BMMCs (One biological replicate of a BMMC culture was derived from 2 wt or 2 *Nbeal2* ^−/−^ mice). We used ImageJ (National Institute of Health, USA) for the quantifications of Western blots. For quantification of protein expressions, the intensities of protein bands were determined and normalised to the respective tubulin controls. For quantification of phosphorylations, the intensities of the phosphorylated protein bands were normalised to the respective values of total protein bands. Thereby, the signal of wt BMMCs (unstimulated) control blots was set as 1.

### 1.13 Statistical Analysis

All experiments were performed at least 3 times (if not otherwise stated). For Western blot or flow cytometry experiments, one representative experiment is shown. For quantification of flow cytometry experiments the summary of at least *n* = 3 biological replicates (if not otherwise stated) are shown and were compared to cells generated from wt mice and/or untreated and/or unstimulated cells. For ELISA experiments we show the summary of results obtained from *n* = 3 biological replicates of wt and *Nbeal2* ^−/−^ BMMCs which were splitted into at least 6 technical replicates per condition. One biological BMMC replicate consists of pooled bone marrow cells from 2 mice. Shown is the mean ± SEM. For statistical analysis we used SigmaPlot 13.0 (Systat Software Inc). To determine the significance between two groups, we used unpaired Students *t*‐test and statistical significance was accepted for *p* ≤ 0.05.

## 2 Introduction

NBEAL2 is a Beige and Chediak‐Higashi domain (BEACH)‐domain containing protein that is predominantly expressed in cells of the haematopoietic system [5]. NBEAL2 contains a BEACH, a concavalin A‐like lectin (ConA), a pleckstrin homology (PH) and a WD40‐repeat (WDR) domain and maintains the stability of α‐granules in several cell types [6, 7, 8]. The localisation of NBEAL2 in different subcellular compartments [9] and the expression of several splice variants indicate that NBEAL2 is involved in diverse cellular processes such as signal transduction, protein sorting and stability. This is underpinned by the fact that NBEAL2 interacts with Dock7, Sec16a, Vac14 [9], STATs [10] and RPS6 [1], proteins that are involved in various biological processes. Due to these multiple functions, NBEAL2 inactivation by mutations leads to dysregulated functions in megakaryocytes, platelets, neutrophils, NK cells and T‐cells [5, 6, 10, 11, 12, 13]. All these dysregulations were identified in patients with the gray platelet syndrome (GPS), a bleeding disorder that is phenotypically characterised by splenomegaly and macrothrombocytopenia [5, 14]. In mice, *Nbeal2* inactivation results in similar defects [6] and, additionally, in multiple functional abnormalities in MCs, which encompass the accumulation of several proteins (e.g., transcription factors and signal transduction proteins), a prolonged growth factor‐independent survival and a strongly increased alarmine‐induced production of pro‐inflammatory cytokines [1, 10]. Nbeal2 also controls the subcellular localisation of proteins. Consequently, Nbeal2 inactivation leads to an increased surface expression of proteins normally residing in intracellular granules such as LAMP‐2 or endocytosed proteins [6, 7, 10]. Given that the multi‐drug‐resistant protein 1 (Mdr‐1) ABCB1 is also located in granules [15], we speculated that Nbeal2 also controls the subcellular localisation of ABCB1 in MCs. In general, ABCB1 belongs to the family of ATP‐binding cassette transporters (ABC transporters), which are transmembrane proteins predominantly located in intracellular compartments with only low expression on the cells surface [16]. In contrast to this, some tumour cell types predominantly express ABCB1 on the cell surface, which leads to resistance to xenobiotics [17, 18]. This increased ABCB1 surface expression can be mediated by different mechanisms such as highly activated MAP‐kinase signalling pathways [19, 20, 21]. We identified a novel mechanism that leads to a strong surface expression of ABCB1. Thereby, inactivation of *Nbeal2* resulted in an excessive surface expression of ABCB1 and thus, to the formation of c‐Kit+/ABCB1+ MCs. Collectively, these findings show that the structural protein Nbeal2 maintains the intracellular localisation of ABCB1, thereby preventing its excessive surface expression on mast cells.

### 2.1 Nbeal2 Regulates the Localisation of ABCB1 in MCs

**FIGURE 1:**
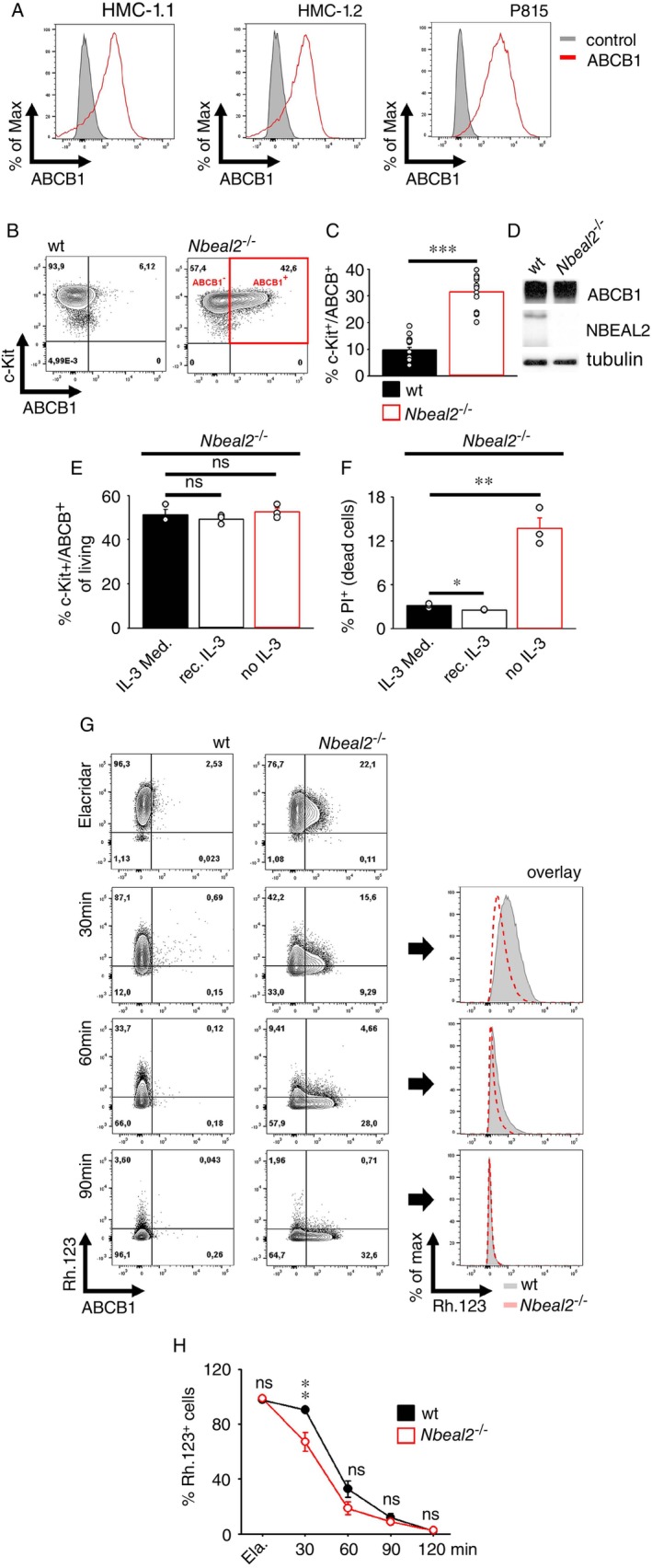
Nbeal2 inactivation stabilises ABCB1 on BMMCs. (A) HMC‐1.1, HMC‐1.2 and P815, were stained for ABCB1 and were analysed by flow cytometry (grey: unstained). (B) Wt and *Nbeal2* ^−/−^ BMMCs were stained for c‐Kit and ABCB1 and were analysed by flow cytometry. (C) Statistical analysis of (B) (means ± SEMs of *n* = 17 biological replicates of wt and *Nbeal2* ^−/−^ BMMCs are shown). (D) Wt and *Nbeal2* ^−/−^ BMMC lysates were analysed by Western blotting. (E, F) *Nbeal2* ^−/−^ BMMCs were either cultured in medium supplemented with IL‐3 from different sources [Medium supplemented with supernatants from IL‐3‐producing cells (IL‐3 Med.) or Medium supplemented with recombinant IL‐3 (rec. IL‐3)] or in medium without IL‐3 (no IL‐3) for 48 h. Subsequently, BMMCs were stained for c‐Kit and ABCB1 in presence of PI and were analysed by flow cytometry. Statistical analyses of c‐Kit+/ABCB1+ *Nbeal2* ^−/−^ BMMCs (E) and of the percentages of dead BMMCs (F) are shown (means ± SEMs of *n* = 3 biological replicates of *Nbeal2* ^−/−^ BMMCs). (G) Rh.123‐loaded wt and *Nbeal2* ^−/−^ BMMCs were stained for ABCB1. After the indicated time points cells were analysed by flow cytometry. (H) Statistical analysis of (G) (means ± SEMs of *n* = 9 biological replicates of wt and *Nbeal2* ^−/−^ BMMCs; the statistics were performed between the groups of the respective time point).

In patients with mast cell leukaemia (MCL), indolent systemic mastocytosis (ISM) and aggressive systemic mastocytosis (ASM), loss of heterozygosity (LOH) of chromosome region 3p21 leads to the loss of 10 genes, among them *NBEAL2* [22]. Interestingly, on the human MCL cell lines HMC‐1.1 and HMC‐1.2, as well as on the murine mastocytoma cell line P815, we detected a strong surface expression of ABCB1 (Figure 1A). *Nbeal2* ^−/−^ BMMCs contain fewer granules, demonstrating that Nbeal2 stabilises granules and facilitates the retention of granule contents [10]. Interestingly, MC granules also contain ABCB1 [15]. Therefore, we speculated a link between *NBEAL2* inactivation and the surface localisation of ABCB1. To determine the role of Nbeal2 in the regulation of ABCB1 localisation, we used MCs generated from wt and *Nbeal2* ^−/−^ mice. Indeed, compared to wt BMMCs, ABCB1 was strongly expressed on the cell surface of *Nbeal2* ^−/−^ BMMCs, leading to the formation of the new c‐Kit+/ABCB1+ population [Figure 1B,C and Figure S1A (staining controls)]. Due to these results, we expected an increased total ABCB1 protein expression in mature *Nbeal2* ^−/−^ BMMCs, leading to strong ABCB1 surface localisation. However, the protein levels of ABCB1 were similar in wt and *Nbeal2* ^−/−^ BMMCs (Figure 1D), showing that Nbeal2 deficiency does not alter the protein level but alters the subcellular localisation of present ABCB1. Next, we wanted to determine the source of the c‐Kit+/ABCB1+ *Nbeal2* ^−/−^ BMMC population. We hypothesised c‐Kit+/ABCB1− *Nbeal2* ^−/−^ BMMCs as the source of the ABCB1+ population. To test this, we sorted mature bulk *Nbeal2* ^−/−^ BMMCs for ABCB1 to obtain c‐Kit+/ABCB1− and c‐Kit+/ABCB1+ BMMCs. Upon further culturing of c‐Kit+/ABCB1‐*Nbeal2* ^−/−^ BMMCs for several days, we detected accumulation of ABCB1 on the cell surface, resulting again in the formation of two BMMC populations (Figure S1B). In contrast to this, the ABCB1 surface expression remained unchanged on sorted c‐Kit+/ABCB1+ *Nbeal2* ^−/−^ BMMCs (Figure S1C). This demonstrated that the c‐Kit+/ABCB1+ *Nbeal2* ^−/−^ BMMCs population arises from c‐Kit+/ABCB1− *Nbeal2* ^−/−^ BMMCs. Next, we wanted to investigate the timing of the surface localisation of ABCB1 on *Nbeal2* ^−/−^ BMMCs during the in vitro differentiation. Therefore, we cultured bone marrow cells from wt or *Nbeal2* ^−/−^ mice and assessed the surface expression of ABCB1 on c‐Kit+ cells. As shown in Figure S1D,E, the ABCB1 surface localisation was detected from the 3rd week of differentiation on *Nbeal2* ^−/−^ BMMCs, showing that the surface expression of ABCB1 is an event mediated during the in vitro differentiation process on c‐Kit+/*Nbeal2* ^−/−^ BMMCs.

### 2.2 IL‐3 Is Not Required for the Localisation of ABCB1 on *Nbeal2* ^−/−^ BMMCs

Differentiation, survival and the expression of several surface molecules in BMMCs depend on IL‐3 [23, 24]. Thus, we tested whether IL‐3, which is present in the differentiation medium of BMMCs, would also be required for the stability of c‐Kit+/ABCB1+ *Nbeal2* ^−/−^ BMMC. Compared to culturing *Nbeal2* ^−/−^ BMMCs in medium with IL‐3 [either conditioned IL‐3‐containing medium (IL‐3‐Med.) or medium supplemented with recombinant IL‐3 (rec. IL‐3)], culturing in media without IL‐3 did not alter the surface expression of ABCB1 (Figure 1E). Thereby, exogenous IL‐3 was functional, as withdrawal of IL‐3 (no IL‐3) induced enhanced cell death in *Nbeal2* ^−/−^ BMMCs (Figure 1F), confirming the typical survival function of IL‐3 on BMMCs. This demonstrated that IL‐3 is not essential for the formation of c‐Kit+/ABCB1+ *Nbeal2* ^−/−^ BMMCs.

Next, we tested the functionality of ABCB1 and compared the efflux rate of Rhodamine 123 (Rh.123), a FITC‐like fluorochrome and hydrophobic ABCB1 substrate [25] from the cytosol in wt and *Nbeal2* ^−/−^ BMMCs. Despite the very low ABCB1 expression, Rh.123 is surprisingly exported very quickly from wt BMMCs (Figure 1G,H). Nevertheless, the comparison of the efflux rate of wt BMMCs and *Nbeal2* ^−/−^ BMMCs showed an even faster export of Rh.123 from *Nbeal2* ^−/−^ BMMCs (Figure 1G,H), demonstrating that *Nbeal2* ^−/−^ BMMCs express more functionally active ABCB1 on their cell surface. To sum up, Nbeal2 preserves BMMCs from an excessive ABCB1 surface expression independently from IL‐3.

### 2.3 Dysregulation of ABCB1 Is Also Detectable on *Nbeal2* ^−/−^ Connective Tissue MCs (CTMCs) and MCs With CRISPR/Cas9‐Mediated *Nbeal2* Deletion

The above‐presented experiments were carried out using BMMCs, which represent immature MCs. In order to investigate the ABCB1 surface expression on fully matured MCs, we used connective tissue MCs (CTMCs) isolated from the peritoneal cavity of wt and *Nbeal2* ^−/−^ mice, that were expanded in the presence of SCF and IL‐3. Similar to *Nbeal2* ^−/−^ BMMCs, *Nbeal2* ^−/−^ CTMCs were also split into c‐Kit+/ABCB1− and c‐Kit+/ABCB1+ cells (Figure S1F), whereas the total protein level of ABCB1 appeared again unchanged (Figure S1G). However, all these data were obtained from animals with a complete absence of *Nbeal2* (*Nbeal2* ^−/−^ mice). Therefore, one could speculate that the strong ABCB1 surface expression on *Nbeal2* ^−/−^ MCs might be a developmental defect occurring during MC differentiation in vivo and in vitro. To investigate the influence of Nbeal2 in mature murine MCs, we deleted *Nbeal2* by CRISPR/Cas9 technology in the murine MC‐line MC/9 [1] which is homologous to in vitro generated wt BMMCs [26, 27]. Whereas the expression of c‐Kit was comparable in parental and *Nbeal2*KO MC/9 cells (Figure S1H), the surface expression of ABCB1 was strongly increased upon *Nbeal2* deletion (Figure S1I). These data demonstrate that the dysregulated distribution of ABCB1 on the cell surface is not a developmental defect but rather a common process triggered by *Nbeal2* deletion in developing or mature MCs.

### 2.4 IL‐33 Downregulates ABCB1 on *Nbeal2* ^−/−^ BMMCs

**FIGURE 2:**
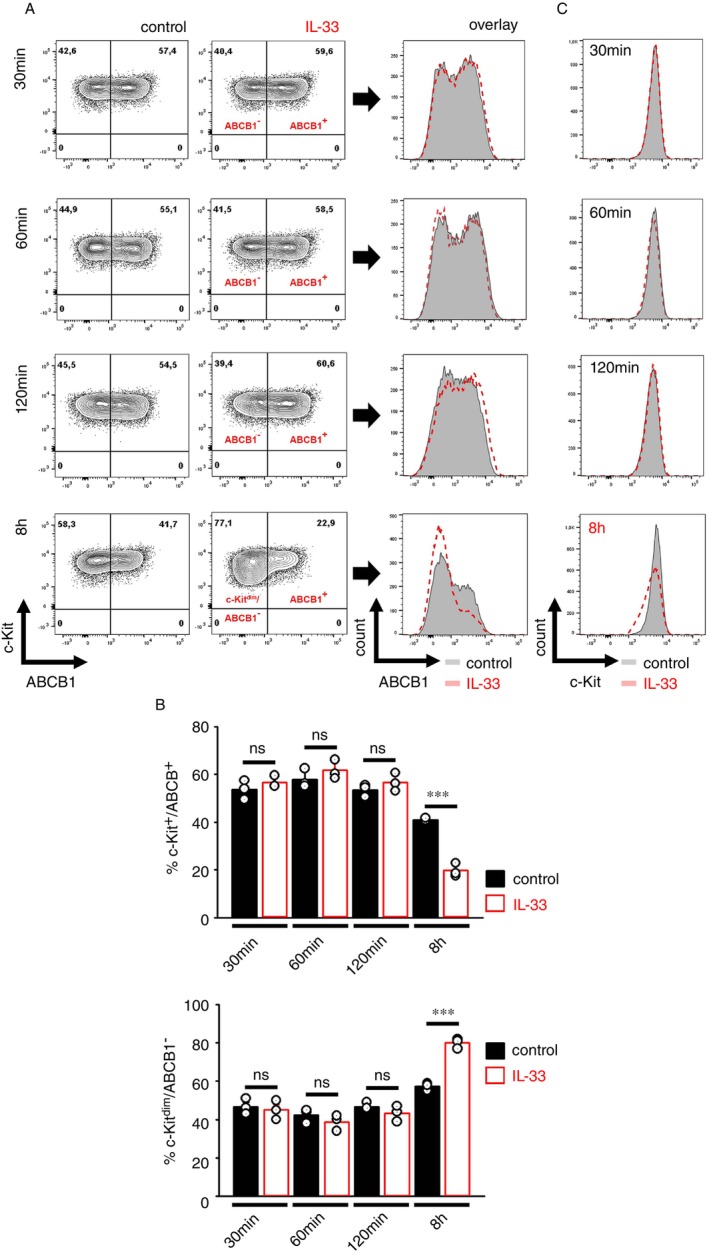
IL‐33 converts ABCB1+ into ABCB1− *Nbeal2* ^−/−^ BMMCs. (A) *Nbeal2* ^−/−^ BMMCs were stimulated with IL‐33 for the indicated time periods until 8 h, were subsequently stained for c‐Kit and ABCB1 and were analysed by flow cytometry. (B) Statistical analysis of (A) (means ± SEMs of *n* = 3 biological replicates of *Nbeal2* ^−/−^ BMMCs). (C) Shown are the histograms (overlay) of c‐Kit from unstimulated and IL‐33‐activated *Nbeal2* ^−/−^ BMMCs.

In general, the surface expression of ABCB1 is modulated by several growth factors and cytokines [28, 29, 30] and the resulting MAP‐kinase activation [19, 31]. MCs strongly express the IL‐33R and are thus highly responsive to the alarmine IL‐33 via the TAK1‐IKK2‐p65/RelA signalling axis [32] as well as MAP‐kinases such as ERK1/2, p38 MAPK and JNK1/2 [33, 34, 35]. Nbeal2 deficiency did not affect the IL‐33‐induced activation of the TAK1‐IKK2‐p65/RelA module [1] but resulted in a stronger activation of the MEK1/2‐ERK1/2 module and of p38 MAPK (Figure S2A), whereas wt and *Nbeal2* ^−/−^ BMMCs equally express the IL‐33R (Figure S2B). Thereby, the MEK1/2‐ERK1/2 module and p38 MAPK are differentially involved in the increased cytokine production (Figure S2C), but despite their stronger activation in *Nbeal2* ^−/−^ BMMCs, the IL‐33‐induced production of eicosanoids was not affected in *Nbeal2* ^−/−^ compared to wt BMMCs (Figure S2D,E). Given that MAP‐kinases are stronger activated by IL‐33 in *Nbeal2* ^−/−^ BMMCs and are known to stabilise ABCB1 [19, 31], we hypothesised that stimulation with IL‐33 would further increase the ABCB1 surface expression, which might contribute to the enhanced cytokine secretion in *Nbeal2* ^−/−^ BMMCs, as we previously described [1]. To test this hypothesis, we used the ABCB1 blocker Elacridar. However, as shown in Figure S2F Elacridar did not affect the IL‐33‐induced cytokine production in wt or *Nbeal2* ^−/−^ BMMCs. Unexpectedly, the presence of IL‐33 for longer periods did not enhance but slowly (after 8 h) (Figure 2A,B) and constantly (until 16 h) (Figure S3A,B) reduced the ABCB1 surface expression. This resulted in the formation of c‐Kit+/ABCB1− *Nbeal2* ^−/−^ BMMCs, which were additionally characterised by diminished c‐Kit surface expression (Figure 2C, after 8 h). Due to these results, IL‐33‐generated c‐Kit+/ABCB1− *Nbeal2* ^−/−^ BMMCs were designated as c‐Kit^dim^/ABCB1− *Nbeal2* ^−/−^ BMMCs. To test whether IL‐33 also modulates the upregulation of ABCB1 surface expression on *Nbeal2* ^−/−^ BMMCs during their in vitro differentiation, we generated *Nbeal2* ^−/−^ BMMCs in the presence of IL‐33. Expectedly, the ABCB1 surface expression was blocked in the presence of IL‐33 on *Nbeal2* ^−/−^ BMMCs (Figure S3C). However, this effect was only transient, as withdrawal of IL‐33 after the 3rd week restored the upregulated ABCB1 expression again (Figure S3D,E). These data showed that the presence of IL‐33 temporarily blocks the ABCB1 surface translocation and thus induces the conversion of c‐Kit+/ABCB1+ into c‐Kit^dim^/ABCB1− *Nbeal2* ^−/−^ BMMCs, which are robust producers of pro‐inflammatory cytokines.

### 2.5 The IL‐33‐Induced Generation of c‐Kit^dim^/ABCB1− *Nbeal2* ^−/−^ BMMCs Depends on the TAK1‐IKK2 Module

**FIGURE 3:**
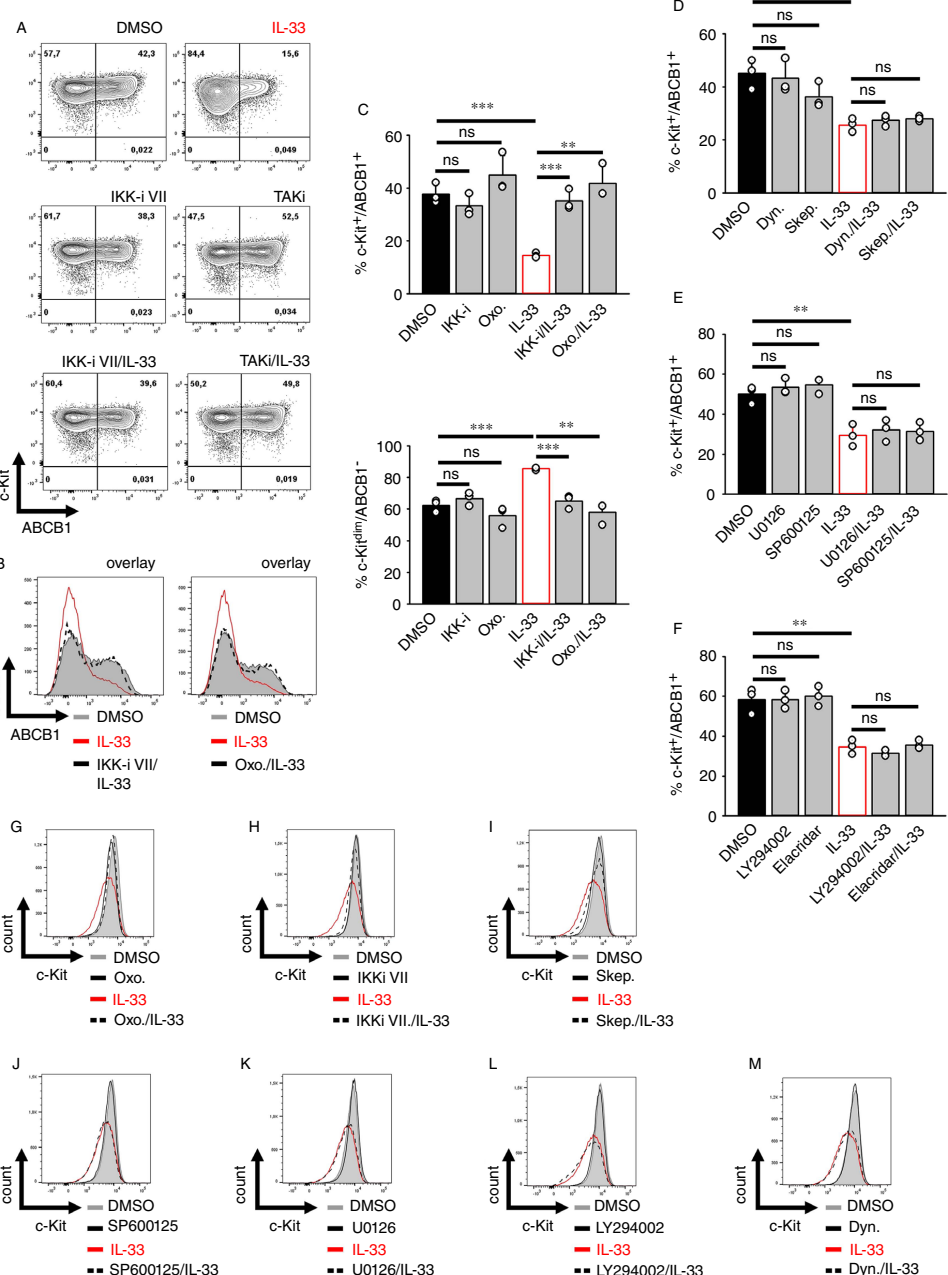
IL‐33 converts ABCB1+ into ABCB1− *Nbeal2* ^−/−^ BMMCs via the TAK1‐IKK2 module. (A–C) Upon treatment with the IKK‐inhibitor VII (1 μM) (IKK‐i), 5Z‐7‐Oxozeanol (5 μM) (Oxo.) or with vehicle (DMSO), *Nbeal2* ^−/−^ BMMCs were stimulated with IL‐33 for 24 h. Cells were stained for c‐Kit and ABCB1 and were analysed by flow cytometry. Representative dot plots are shown in (A). Overlays of representative data from (A) are shown in (B). (C) Statistical analysis of (A) (means ± SEMs of *n* = 3 biological replicates of *Nbeal2* ^−/−^ BMMCs). (D–F) *Nbeal2* ^−/−^ BMMCs were treated with vehicle (DMSO, as indicated in the figure) or either with Dynasore (10 μM) (Dyn.), or L‐Skepinone (5 μM) (Skep.) (D); or U0126 (10 μM), or SP600125 (5 μM) (E); or LY294002 (5 μM), or Elacridar (5 μM) (F). 30 min later, cells were stimulated with IL‐33 (50 ng/mL) for 24 h. Cells were then stained for c‐Kit and ABCB1 and were analysed by flow cytometry (Shown are the means ± SEMs of *n* = 3 biological replicates of *Nbeal2* ^−/−^ BMMCs). (G–I) *Nbeal2* ^−/−^ BMMCs were treated with vehicle (DMSO, as indicated in the figure) or either with 5Z‐7‐Oxozeanol (5 μM) (Oxo.) (G), or the IKK‐inhibitor VII (1 μM) (IKKi) (H), or L‐Skepinone (5 μM) (Skep.) (I). 30 min later, cells were stimulated with IL‐33 (50 ng/mL) for 24 h and were stained for c‐Kit and ABCB1 for flow cytometry analysis. Shown are representative overlays from *n* = 3, biological replicates of *Nbeal2* ^−/−^ BMMCs. (J–M) *Nbeal2* ^−/−^ BMMCs were treated with vehicle (DMSO, as indicated in the figure) or either with SP600125 (5 μM) (J), or U0126 (10 μM) (K), or LY294002 (5 μM) (L), or Dynasore (10 μM) (M). 30 min later, cells were stimulated with IL‐33 (50 ng/mL) for 24 h. Cells were then stained for c‐Kit and ABCB1 and analysed by flow cytometry. Shown are representative overlays from *n* = 3, biological replicates of *Nbeal2* ^−/−^ BMMCs.

To identify the signalling pathways mediating the IL‐33‐induced downregulation of surface ABCB1 on *Nbeal2* ^−/−^ BMMCs, we targeted established IL‐33‐activated signalling pathways by using pharmacological inhibitors. To block the TAK1‐IKK2 module, we employed the TAK1 inhibitor 5Z‐7‐oxozeanol (Oxo.) and the IKK‐inhibitor VII (IKK‐i). Whereas inhibition of TAK1 or IKK2 did not influence the high basal surface expression of ABCB1 in resting *Nbeal2* ^−/−^ BMMCs, these inhibitors prevented the IL‐33‐induced downregulation of surface ABCB1 on c‐Kit+ *Nbeal2* ^−/−^ BMMCs (Figure 3A–C). Downstream of the TAK1‐IKK2 module, IL‐33 activates PI3Ks, ERK1/2, p38 MAPK and JNK1/2 [32, 33, 34] and mediates internalisation processes [36] which might be dynamine‐dependent. However, none of the treatments with either Dynasore (Dyn.) (dynamine inhibitor), L‐Skepinone (p38 MAPK inhibitor) (Skep.) (Figure 3D), U0126 (ERK1/2 activation inhibitor), SP600125 (JNK1/2 inhibitor) (Figure 3E), LY294002 (PI3K inhibitor), or Elacridar (Figure 3F) did neither affect the ABCB1 surface expression on resting nor reversed the IL‐33‐stimulated ABCB1 downregulation on *Nbeal2* ^−/−^ BMMCs.

As described above, IL‐33 also downregulated c‐Kit's surface expression, leading to the formation of c‐Kit^dim^/ABCB1− *Nbeal2* ^−/−^ BMMCs. To investigate how IL‐33 mediates this effect, we again inhibited the TAK1‐IKK2 module and thereby could reverse the IL‐33‐induced c‐Kit downregulation (Figure 3G,H). The same effect was observed after inhibition of p38 MAPK (Figure 3I) but not after inhibition of JNK1/2, ERK1/2, PI3K, or dynamine (Figure 3J–M). These data show that the IL‐33‐induced downregulation of the surface localisation of c‐Kit and ABCB1 depends on the TAK‐IKK2 module, whereas the downregulation of c‐Kit is additionally mediated by p38 MAPK in *Nbeal2* ^−/−^ BMMCs.

### 2.6 The Opposite Roles of Tyrosine Kinases in Resting and IL‐33‐Activated *Nbeal2* ^−/−^ BMMCs

**FIGURE 4:**
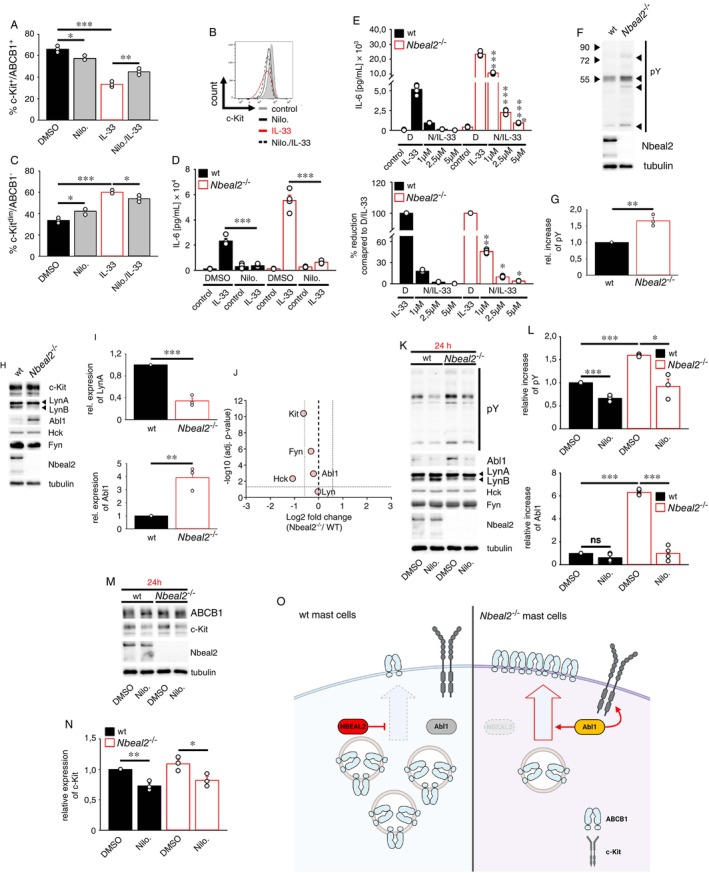
Nilotinib destabilises accumulated Abl1 and reduces the ABCB1 surface localisation in *Nbeal2* ^−/−^ BMMCs. (A–C) *Nbeal2* ^−/−^ BMMCs were treated with vehicle (DMSO, as indicated in the figure) or with Nilotinib (5 μM) (Nilo.). After stimulation with IL‐33 (50 ng/mL) for 24 h, cells were stained for c‐Kit and ABCB1 and analysed by flow cytometry. Statistical analysis is shown in (A, C) (means ± SEMs of *n* = 3 biological replicates of wt and *Nbeal2* ^−/−^ BMMCs). Representative histograms of c‐Kit staining of *Nbeal2* ^−/−^ BMMCs are shown in (B). (D) Wt and *Nbeal2* ^−/−^ BMMCs were treated with vehicle (DMSO, as indicated in the figure) or with Nilotinib (5 μM) (Nilo.). Statistical analysis is shown (means ± SEMs of *n* = 4 biological replicates of wt and *Nbeal2* ^−/−^ BMMCs). (E) Wt and *Nbeal2* ^−/−^ BMMCs were treated with increasing concentrations of vehicle (DMSO shown as D) or with Nilotinib (shown as N) (1, 2.5, or 5 μM). 30 min later, cells were stimulated with IL‐33 (50 ng/mL) for 24 h and supernatants were analysed by ELISA. (E; upper panel) Statistical analysis is shown (means ± SEMs of *n* = 4 biological replicates of wt and *Nbeal2* ^−/−^ BMMCs; the statistics were performed between the groups of the respective treatments in wt and *Nbeal2* ^−/−^ BMMCs). (E; lower panel) Shown is the Nilotinib‐mediated reduction of the IL‐33‐induced IL‐6 production. Thereby, the IL‐6 production in the presence of IL‐33 (without Nilotinib, shown as N) in wt and *Nbeal2* ^−/−^ BMMCs was set as 100% (means ± SEMs of *n* = 4 biological replicates of wt and *Nbeal2* ^−/−^ BMMCs; the statistics were performed between the groups of the respective treatments in wt and *Nbeal2* ^−/−^ BMMCs). (F) Lysates of wt and *Nbeal2* ^−/−^ BMMCs were analysed by Western blotting. Representative Western blots are shown. (G) Statistical analysis of densitometric analysis of (F) is shown (means ± SEMs of *n* = 3 biological replicates of wt and *Nbeal2* ^−/−^ BMMCs). (H) Lysates of wt and *Nbeal2* ^−/−^ BMMCs were analysed by Western blotting. Representative Western blots are shown. (I) Statistical analysis of densitometric analysis of (H) is shown (means ± SEMs of *n* = 3 biological replicates of wt and *Nbeal2* ^−/−^ BMMCs). (J) Changes in transcript levels of selected genes from wt and *Nbeal2* ^−/−^ BMMC RNA_seq_ data (*n* = 4 biological replicates of wt and *Nbeal2* ^−/−^ BMMCs). Genes with a −Log10 (adjusted *p*‐value) of 1.3 (*p* ≤ 0.05) were assessed as differentially expressed genes (DEGs). (K–N) Wt and *Nbeal2* ^−/−^ BMMCs were treated with vehicle (DMSO, as indicated in the figure) or with Nilotinib (5 μM) for 24 h. Lysates were analysed by Western blotting. Representative Western blots are shown. (L, N) Statistical analysis of densitometric analysis of (K, M) is shown [means ± SEMs of *n* = 3 (for Abl1, *n* = 4) biological replicates of wt and *Nbeal2* ^−/−^ BMMCs]. (O) In wt BMMCs, Nbeal2 controls the surface expression of ABCB1. In *Nbeal2* ^−/−^ BMMCs, Abl1 is stabilised and supports the surface expression of ABCB1 and c‐Kit. (O) Was created by using Biorender.

*Nbeal2* ^−/−^ BMMCs share some basic features with tumour cells. However, we could not determine the detailed mechanism which results in the extensive surface expression of ABCB1 on resting *Nbeal2* ^−/−^ BMMCs. Tyrosine kinases also stabilise ABCB1 in tumour cells [37]. So, we determined the role of tyrosine kinases in the upregulation of the ABCB1 surface expression on resting *Nbeal2* ^−/−^ BMMCs. For this, we used the tyrosine kinase inhibitor Nilotinib, which is described to inhibit several tyrosine kinases [38, 39]. Unexpectedly, Nilotinib (Nilo.) alone reduced both the ABCB1 and c‐Kit surface expression on resting *Nbeal2* ^−/−^ BMMCs, albeit to a lesser extent than the IL‐33 stimulation (Figure 4A,B). As a consequence of this, the frequencies of c‐Kit^dim^/ABCB1− *Nbeal2* ^−/−^ BMMCs slightly increased (Figure 4C). Next, we determined the effect of Nilotinib on IL‐33‐activated *Nbeal2* ^−/−^ BMMCs. Controversially, Nilotinib treatment reversed the IL‐33‐induced downregulation of ABCB1 on c‐Kit+/ABCB1+ *Nbeal2* ^−/−^ BMMCs (Figure 4A,B) and blocked the resulting cytokine response (Figure 4D) more efficiently in wt compared to *Nbeal2* ^−/−^ BMMCs (Figure 4E). These data indicated opposite roles of tyrosine kinases in resting and IL‐33‐activated *Nbeal2* ^−/−^ BMMCs. Whereas tyrosine kinases support the surface expression of c‐Kit and ABCB1 on resting c‐Kit+/ABCB1+ *Nbeal2* ^−/−^ BMMCs, they downregulate c‐Kit and ABCB1 in the presence of IL‐33 and thus mediate the formation of cytokine producing c‐Kit^dim^/ABCB1− *Nbeal2* ^−/−^ BMMCs.

### 2.7 Stabilised Abl1 Supports the ABCB1 Surface Localisation on Resting *Nbeal2* ^−/−^ BMMCs

Next, we aimed to determine the mechanisms leading to the opposite roles of tyrosine kinases in resting and IL‐33‐activated *Nbeal2* ^−/−^ BMMCs regarding the ABCB1 surface expression. We started to determine the mechanism in resting *Nbeal2* ^−/−^ BMMCs. We hypothesised enhanced tyrosine kinase activities, which mediate the Nilotinib‐sensitive ABCB1 and c‐Kit surface expression on resting *Nbeal2* ^−/−^ BMMCs. To indirectly determine tyrosine kinase activities in wt and *Nbeal2* ^−/−^ BMMCs, we performed Western blot experiments directed against pan‐tyrosine phosphorylations (pYs). First, we compared the pYs of resting wt and *Nbeal2* ^−/−^ BMMCs. Indeed, Nbeal2 deficiency resulted in increased pYs (Figure 4F,G), indicating enhanced tyrosine kinase activities. As Nbeal2 inactivation was shown to result in intracellular accumulations of various proteins [1, 10], we speculated that tyrosine kinases might as well be stabilised. As shown in Figure 4H,I, Nbeal2 deficiency stabilised Abl1 but destabilised the splice variant of the src‐family kinase (SFK) Lyn, which is named as LynB. However, the expression of c‐Kit and of the SFKs LynA, Hck, as well as Fyn remained unaffected in *Nbeal2* ^−/−^ BMMCs. Given that neither Abl1 nor Lyn were altered on the transcript level when we analysed the differentially expressed genes (DEGs) between *Nbeal2* ^−/−^ and wt cells (Figure 4J), this suggests that Nbeal2 influences the protein stability of Abl1 and Lyn. Abl1 is a known target of Nilotinib [40] and therefore, we tested the effect of Nilotinib on the increased pY in *Nbeal2* ^−/−^ BMMCs. Importantly, Nilotinib reduced the increased pYs and surprisingly destabilised Abl1 but did not affect the stability of the SFKs LynA, Hck and Fyn in *Nbeal2* ^−/−^ BMMCs (Figure 4K,L). This data show a correlation between Abl1 stabilisation, the increased pYs and importantly the increased surface expression of ABCB1 in *Nbeal2* ^−/−^ BMMCs. Therefore, we believe that accumulated Abl1 mediates the increased pYs and the stabilisation of ABCB1 on the cell surface of *Nbeal2* ^−/−^ BMMCs. Next, we examined the effect of Nilotinib on the total protein level of ABCB1 and c‐Kit, which were both downregulated from the surface by Nilotinib treatment. Whereas Nilotinib treatment decreased the total levels of c‐Kit, the total levels of ABCB1 remained unchanged (Figure 4M,N). This suggests that the Nilotinib‐sensitive decrease of c‐Kit on the cell surface is caused by a general protein destabilisation, whereas the change in the ABCB1 surface expression is a result of a block in translocation, keeping ABCB1 internalised. Together, these data indicate that Nbeal2 deficiency in combination with stabilised Abl1 maintains resting c‐Kit+/ABCB1+ BMMCs (Figure 4O).

### 2.8 IL‐33 Downregulated ABCB1 by SFKs

**FIGURE 5:**
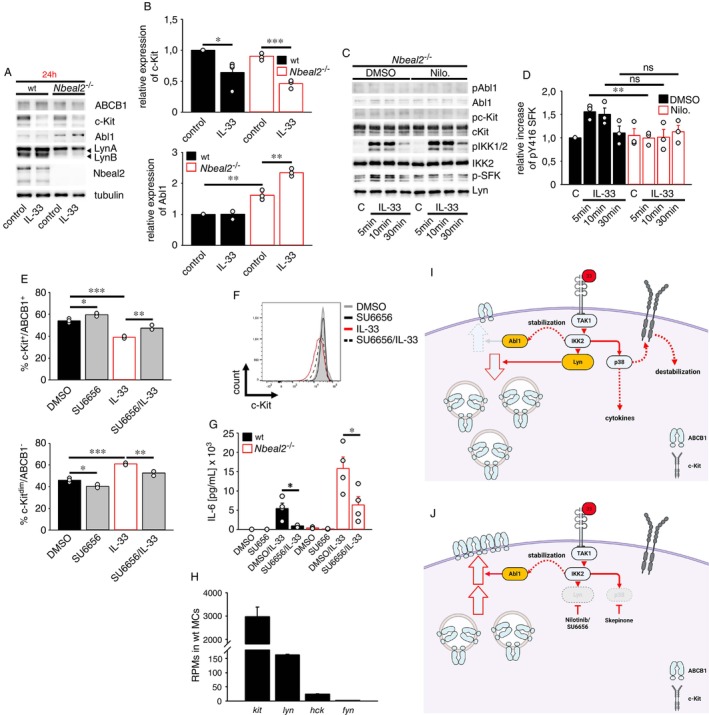
IL‐33 destabilises ABCB1 via Src‐family kinases (SFKs). (A) Wt and *Nbeal2* ^−/−^ BMMCs were stimulated with IL‐33 (50 ng/mL) for 24 h. Lysates were analysed by Western blotting. Representative blots are shown. (B) Statistical analyses of densitometric analysis of (A) are shown [means ± SEMs of *n* = 3 (for c‐Kit, *n* = 4) biological replicates of wt and *Nbeal2* ^−/−^ BMMCs]. (C) Wt and *Nbeal2* ^−/−^ BMMCs were stimulated with IL‐33 (50 ng/mL) as indicated. Lysates were analysed by Western blotting. Representative blots are shown. (D) Statistical analysis of densitometric analysis of (C) are shown (means ± SEMs of *n* = 3 biological replicates of wt and *Nbeal2* ^−/−^ BMMCs). (E) *Nbeal2* ^−/−^ BMMCs were treated vehicle (DMSO, as indicated in the figure) or with SU6656 (5 μM). 30 min later, cells were stimulated with IL‐33 (50 ng/mL) for 24 h and were stained for c‐Kit and ABCB1. Cells were analysed by flow cytometry and statistical analysis of summarised data is shown (means ± SEMs of *n* = 3 biological replicates of *Nbeal2* ^−/−^ BMMCs). (F) Representative histogram of the c‐Kit staining of *Nbeal2* ^−/−^ BMMCs is shown (*n* = 3 biological replicates). (G) Wt and *Nbeal2* ^−/−^ BMMCs were treated with vehicle (DMSO, as indicated in the figure) or with SU6656 (5 μM). 30 min later, cells were stimulated with IL‐33 (50 ng/mL) for 24 h and supernatants were analysed by ELISA. Statistical analysis of summarised data is shown [means ± SEMs of *n* = 4 (for ELISA) biological replicates of wt and *Nbeal2* ^−/−^ BMMCs]. (H) The expressions of mRNA transcripts (reads per million, RPMs) of selected tyrosine kinases in wt BMMCs are summarised (mean ± SEMs of *n* = 4 biological replicates). (I) In IL‐33‐activated *Nbeal2* ^−/−^ BMMCs, Abl1 is further stabilised and the activation of Lyn and p38 is induced. Whereas, activated p38 supports c‐Kit destabilisation, activated Lyn overcomes the stabilising effect of Abl1 and thus mediates the internalisation of ABCB1. (J) Whereas treatment of *Nbeal2* ^−/−^ BMMCs with Nilotinib or SU6656 blocked SFKs (probably Lyn), Skepinone inhibits p38. The immediate inhibition of Lyn by Nilotinib or SU6656 results in a Abl1‐mediated re‐surface expression of ABCB1 whereas Inhibition of p38 normalised the c‐Kit surface expression on *Nbeal2* ^−/−^ BMMCs again. (I, J) Were created by using Biorender.

Next, we determined the mechanism resulting in a Nilotinib‐sensitive and IL‐33‐induced downregulation of ABCB1 and c‐Kit in *Nbeal2* ^−/−^ BMMCs. We found that treatment with Nilotinib alone decreased Abl1, which correlated with a diminished surface expression of ABCB1 on *Nbeal2* ^−/−^ BMMCs. This led to the hypothesis that the stability of Abl1 supports the stability of ABCB1 and c‐Kit on the cell surface of *Nbeal2* ^−/−^ BMMCs (Figure 4O). Stimulation with IL‐33 decreased the ABCB1 surface expression as well, which prompted us to test whether IL‐33 stimulation of *Nbeal2* ^−/−^ BMMCs would also induce a destabilisation of Abl1. Unexpectedly, IL‐33 stimulation of *Nbeal2* ^−/−^ BMMCs for 24 h slightly increased the expression of Abl1 and decreased the total protein level of c‐Kit, whereas ABCB1 and LynA remained unaffected (Figure 5A,B). This indicated that Abl1 is most likely not involved in the IL‐33‐induced downregulation of ABCB1 and c‐Kit in response to IL‐33. Since the downregulation of ABCB1 by IL‐33 is an induced process, we speculated that these events are determined by signalling pathways, which are transiently activated by IL‐33 (until 30 min) in *Nbeal2* ^−/−^ BMMCs. As shown in Figure 5C, IL‐33 stimulations (until 30 min) did neither induce the activation of c‐Kit nor of Abl1 but activated the typical IKK2 pathway and SFKs. Interestingly, the presence of Nilotinib did not influence the basal but blocked the IL‐33‐induced activation of SFKs (Figure 5C,D), whereas the activation and stability of c‐Kit, Abl1 and IKK2 remained unaffected. This indicated that activated SFKs are Nilotinib off‐targets in BMMCs, which could explain the effect of the IL‐33‐induced but Nilotinib‐sensitive downregulation of ABCB1 in *Nbeal2* ^−/−^ BMMCs. To determine the role of SFKs in the IL‐33‐induced downregulation of ABCB1 by using the SFK‐specific inhibitor SU6656 [41]. SU6656 slightly upregulated the basal level of surface ABCB1 and reversed the IL‐33‐induced downregulation of surface ABCB1 but not of c‐Kit (Figure 5E,F). In line with this reduction, SU6656 also suppressed the IL‐33‐induced cytokine response (Figure 5G). Based on these data, we conclude that IL‐33‐activated SFKs [probably Lyn which is the highest expressed and negatively acting SFK in BMMCs (Figure 5H)] overcomes the increased ABCB1 surface expression supported by stabilised Abl1 and thus downregulates ABCB1 from the cell surface (Figure 5I). As an off‐target effect, Nilotinib immediately inhibits IL‐33 transiently activated SFKs. This prevents the negative role of SFKs (most likely Lyn) on the ABCB1 surface expression and restores the Abl1‐supported ABCB1 surface expression on *Nbeal2* ^−/−^ BMMCs (Figure 5J).

## 3 Discussion

We and others found that Nbeal2 deficiency destabilises granules [7, 9] and increases protein stabilities [1, 10] which results in an enhanced survival and an increased production of pro‐inflammatory cytokines in MCs [1]. This implies that Nbeal2 prevents the development of abnormal MCs phenotypes [1]. Here, we show that *Nbeal2* deletion also unleashes the ABCB1 surface expression, resulting in separation of *Nbeal2* ^−/−^ MCs into c‐Kit+/ABCB1− and c‐Kit+/ABCB1+ MC subpopulations, which leads to a faster efflux rate of ABCB1 substrates from *Nbeal2* ^−/−^ compared to wt BMMCs. However, despite the very low surface localisation of ABCB1 in wt BMMCs, the ABCB1 substrate Rh.123 is apparently quickly exported. Interestingly, ABCB1 can also be located in lysosomal structures [42] and rhodamines are quickly inactivated by low pH values [43]. Thus, due to the intact intracellular compartments in wt BMMCs, we speculate that Rh.123 is transported via ABCB1 into lysosomes. This inactivates Rh.123 in the acidic lysosomal environment, which prevents its detection by flow cytometry. Consequently, this pretends a fast but actually non‐existing release of Rh.123 from wt BMMCs. Nevertheless, our data show that Nbeal2 inactivation results in a dysregulated surface localisation of functional ABCB1, which is initiated in the 3rd week of in vitro BMMC differentiation, when in wt BMMCs the granule formation takes place. Given that Nbeal2 is essential for granule formation and retention [7], we speculate that *Nbeal2* ^−/−^ MCs are unable to stabilise ABCB1‐loaded granules within the cell since the granule formation is defective. This results in a permanent surface expression of ABCB1 on *Nbeal2* ^−/−^ MCs, which is normally stored in granules within wt MCs [15].

The investigation of the underlying mechanism revealed that the ABCB1 surface expression on resting *Nbeal2* ^−/−^ BMMCs was sensitive to Nilotinib treatment. The Nilotinib‐mediated destabilisation of surface ABCB1 correlated with a reduction of stabilised Abl1 in *Nbeal2* ^−/−^ BMMCs. This suggested that stabilised Abl1 supports the surface expression of ABCB1 on resting c‐Kit+/ABCB1+ *Nbeal2* ^−/−^ BMMCs. How Nbeal2 could control the stability of Abl1 is unknown. We recently found that Nbeal2 deficiency stabilises RPS6 as well [1] which indicates that Nbeal2 controls the homeostatic RPS6 stability. We reported that Nbeal2, via its WDR domain, interacts with RPS6, which is suggested to be the prerequisite to deliver RPS6 to the ubiquitin‐ligase‐dependent proteasomal degradation pathway [1]. We postulate a similar mechanism for Abl1. In a model predicted by using Boltz‐1 (AlphaFold3) (at: https://neurosnap.ai), we could show that the WDR domain of NBEAL2 can interact with the SH2 domain and parts of the kinase domain of Abl1 (Figure S4A–C). Therefore, we suggest that Nbeal2, together with a yet unidentified ubiquitin‐ligase complex, also controls the homeostatic level of Abl1 via the proteasomal degradation pathway in MCs (Figure S4D).

Not only Nilotinib, but also IL‐33 downregulated the ABCB1 surface expression on *Nbeal2* ^−/−^ BMMCs. Intriguingly, this IL‐33‐induced downregulation of the ABCB1 surface expression and the resulting reduction on *Nbeal2* ^−/−^ BMMCs was not only accompanied by an increase of Abl1, but was also reversed by Nilotinib treatment. Given that Nilotinib targets Abl1, these data would indicate two opposing roles of accumulated Abl1 in association with the surface expression of ABCB1: (i) a stabilising role of Abl1 in resting Kit+/ABCB1+ *Nbeal2* ^−/−^ BMMCs and (ii) a destabilising role of Abl1 in IL‐33‐activated Kit+/ABCB1+ *Nbeal2* ^−/−^ BMMCs. This contradiction prompted us to speculate that stimulation of *Nbeal2* ^−/−^ BMMCs with IL‐33 induced the activation of a mechanism that is inhibited by an off‐target effect of Nilotinib. Indeed, Nilotinib untypically inhibited the IL‐33‐induced activation of SFKs. MCs express many SFKs among them the regulatory SFK Lyn, which is the highest expressed SFK in MCs [44, 45]. Therefore we hypothesise that IL‐33 activates SFKs (e.g., Lyn), which is unintentionally targeted by Nilotinib and as expected by the SFK inhibitor SU6656, to overcome the stabilising effect of Abl1 on the ABCB1 surface expression on *Nbeal2* ^−/−^ BMMCs. How Nilotinib inhibits the IL‐33‐induced activation of SFKs (e.g., Lyn) is currently unknown. Given that Nilotinib does not target SFKs (e.g., Lyn) [46], we assume a yet unidentified target of Nilotinib which acts as an upstream activator for SFKs (e.g., Lyn) in MCs. Inhibition of this target indirectly blocks Lyn activation which results in an Abl1‐dependent re‐expression of ABCB1. Consequently, activated SFKs (e.g., Lyn) downregulate ABCB1 in *Nbeal2* ^−/−^ BMMCs and this points to an antagonistic relationship between stabilised Abl1 and activated SFKs. Whereas Abl1 stabilises ABCB1 on the surface of *Nbeal2* ^−/−^ BMMC, IL‐33‐activated SFKs (e.g., Lyn) have the opposite effect. In summary, our data show that *Nbeal2* deletion results in the formation of c‐Kit+/ABCB1+ *Nbeal2* ^−/−^ BMMCs whose stability is balanced by Abl1 and the IL‐33‐induced activation of SFKs (e.g., Lyn). Whereas Abl1 contributes to the stability of resting Kit+/ABCB1+ *Nbeal2* ^−/−^ BMMCs, IL‐33 destabilised the c‐Kit+/ABCB1+ *Nbeal2* ^−/−^ BMMCs by an SFK‐dependent mechanism.

The relevance of our data is underscored by the fact that the *NBEAL2* gene seems to be inactivated in mastocytosis or MCL patients [22]. Interestingly, on MCL cell lines, we found a strong surface expression of ABCB1 [22] supporting the existence of a link between *NBEAL2* inactivation and the ABCB1 upregulation on the cell surface in human MC neoplasms. Moreover, in many cancer diseases such as acute myeloid leukaemia (AML), damaging and therefore inactivating *NBEAL2* mutations were identified [47, 48]. Given that in AML patients the ABCB1 expression is linked to a worse prognosis [49], we hypothesise that *NBEAL2* deletion or inactivating *NBEAL2* mutations could be a prognostic marker for dysregulated ABCB1 on the cell surface and the resulting tolerance development of cancer cells to chemotherapeutic treatments.

## 4 Conclusion

Our data show that Nbeal2 negatively controls the stability of Abl1 and the ABCB1 cell surface localisation, as *Nbeal2* inactivation leads to a strong Abl1‐supported ABCB1 cell surface localisation on MCs. In contrast to this, the IL‐33‐induced SFK activation antagonises this elevated ABCB1 cell surface localisation on MCs. As high ABCB1 surface levels are correlated with an oncogenic phenotype, our data show that *Nbeal2* inactivation supports the development of MC neoplasms and probably also other cancer entities.

## Author Contributions

R.M., N.A. and J.D. designed research, performed experiments, analysed data. J.D. and N.A. edited the manuscript. P.M.J., C.K., R.H., K.H. and M.G. performed experiments. B.N. and O.W. provided essential reagents. S.D. developed the concept, designed the research, performed experiments, analysed data, made the figures, drafted, wrote and edited the manuscript.

## Ethics Statement

Organs Isolations or the isolation of cells from mice was approved by the Thüringer Landesamt für Lebensmittelsicherheit und Verbraucherschutz (TLLV); Bad Langensalza; licence number of Institute of Immunology, Jena is twz‐36‐2017.

## Consent

The authors have nothing to report.

## Conflicts of Interest

The authors declare no conflicts of interest.

## Supporting information


**Figure S1:** Nbeal2 deficiency upregulates the surface expression of ABCB1. (A) Wt and *Nbeal2* ^−/−^ BMMCs were either stained for c‐Kit alone (FMO for ABCB1 staining) or for c‐Kit and ABCB1. Cells were analysed by flow cytometry. FACS plots and histogram overlays are shown. (B, C) *Nbeal2* ^−/−^ BMMCs were sorted for ABCB1. ABCB1− (B) and ABCB1+ (C) *Nbeal2* ^−/−^ BMMCs were cultured for 3 or 7 days, were subsequently stained for ABCB1 and were analysed by flow cytometry. Overlays are shown. (D) Bone marrow cells from wt and *Nbeal2* ^−/−^ mice were cultured in IL‐3 medium. After the indicated time points cells were stained for c‐Kit and ABCB1 and were analysed by flow cytometry. Representative FACS plots are shown. (E) Statistical analysis of (D) is shown (means ± SEMs of *n* = 15 biological replicates of wt and *Nbeal2* ^−/−^ BMMCs). (F) Wt and *Nbeal2* ^−/−^ CTMCs were stained for c‐Kit and ABCB1 and were analysed by flow cytometry. Flow cytometry plots and overlay of the ABCB1 expression of wt and *Nbeal2* ^−/−^ CTMCs are shown. (G) Western blotting of lysates of wt and *Nbeal2* ^−/−^ CTMCs are shown. (H, I) Parental and *Nbeal2*KO MC/9 cells were stained for c‐Kit and ABCB1 and were analysed by flow cytometry. Histograms of the ABCB1 expression are shown.

**Figure S2:** Nbeal2 deficiency enhanced the IL‐33‐induced production of pro‐inflammatory cytokines via p38. (A) Wt and *Nbeal2* ^−/−^ BMMCs were stimulated with IL‐33 (50 ng/mL) as indicated. Lysates were analysed by Western blotting. Statistical analysis of densitometric analysis are shown below each representative Western blots [means ± SEMs of *n* = 3 (for pJNK1/2, *n* = 4) biological replicates of wt and *Nbeal2* ^−/−^ BMMCs; the statistics were performed between the respective time points in the wt and the *Nbeal2* ^−/−^ groups]. (B, upper panel) Wt and *Nbeal2* ^−/−^ BMMCs were stained for the IL‐33R and were analysed by flow cytometry. Shown is one representative flow cytometry experiment out of *n* = 3 biological replicates of wt and *Nbeal2* ^−/−^ BMMCs. (B, lower panel) Wt and *Nbeal2* ^−/−^ BMMCs were lysed and analysed by Western blotting. Shown are representative Western blots from *n* = 4 biological replicates of wt and *Nbeal2* ^−/−^ BMMCs. (C) Wt and *Nbeal2* ^−/−^ BMMCs were treated with vehicle (DMSO, as indicated) or either with U0126 (10 μM), or L‐Skepinone (5 μM) (Skep.). 30 min later, cells were stimulated with IL‐33 (50 ng/mL) for 24 h and supernatants were analysed by ELISA. Statistical analyses are shown (means ± SEMs of *n* = 4 biological replicates of wt and *Nbeal2* ^−/−^ BMMCs). (D, E) Wt and *Nbeal2* ^−/−^ BMMCs were stimulated with IL‐33 (50 ng/mL) for 24 h. Lipid mediators were extracted from the supernatant by solid‐phase extraction and analysed by UPLC‐MS/MS. Summarising overview is shown in the table (D) and the statistical analysis is shown in (E) (means ± SEMs of *n* = 10 biological replicates of *Nbeal2* ^−/−^ BMMCs). (F) Wt and *Nbeal2* ^−/−^ BMMCs were treated with vehicle (DMSO, as indicated) or with Elacridar (5 μM) (Ela.). 30 min later, cells were stimulated with IL‐33 (50 ng/mL) for 24 h. Supernatants were analysed by ELISA and statistical analyses are shown (means ± SEMs of *n* = 4 biological replicates of wt and *Nbeal2* ^−/−^ BMMCs).

**Figure S3:** IL‐33 reversibly regulates the ABCB1 expression on *Nbeal2* ^−/−^ BMMCs. (A, B) *Nbeal2* ^−/−^ BMMCs were stimulated with IL‐33 (50 ng/mL) for the indicated time periods. Subsequently, cells were stained for c‐Kit and ABCB1 and were analysed by flow cytometry. Representative plots and histogram overlays are shown. (B) Statistical analysis of (A) are shown in (means ± SEMs of *n* = 3 biological replicates of *Nbeal2* ^−/−^ BMMCs). (C–E) Bone marrow cells from wt and *Nbeal2* ^−/−^ mice were either cultured in IL‐3 medium (no IL‐33) or in medium containing IL‐3 and IL‐33 (with IL‐33). After 3 weeks, cells were stained for c‐Kit and ABCB1 and were analysed by flow cytometry. Representative plots are shown (C). (D) From the 3rd week on, IL‐33 was withdrawn from the cell culture and cells remained in basic culture conditions (with IL‐3 but without IL‐33) for another week. After the 4th week, cells were stained for c‐Kit and ABCB1 and were analysed by flow cytometry. Representative plots are shown. (E) Statistical analysis of (C, D) are shown (means ± SEMs of *n* = 3 biological replicates of wt and *Nbeal2* ^−/−^ BMMCs).

**Figure S4:** The proposed model for the NBEAL2/ABL1 interaction and the proposed model for the Nbeal2‐mediated Abl1 degradation in MCs. (A) The interaction of the WDR domain of NBEAL2 (AA 2371–AA 2754) with ABL1 (AA 60–AA 500) was predicted by using Boltz‐1 (AlphaFold3) at: https://neurosnap.ai. Thereby, we obtained 5 possible interaction structures models with a high confidence each. These 5 structures models were merged by using the pymol software. The ABL1 structure obtained by Boltz‐1 (AlphaFold3) is similar to the structure obtained by x‐ray diffraction analysis of ABL1 [1]. (B, C) One of the 5 interaction structure models was randomly chosen and is shown in a mesh/ribbon (B) and ribbon (C) presentation. (D) (1) In MCs, we propose that Abl1 interacts with the WDR domain from Nbeal2. This Nbeal2/Abl1 complex interacts with a yet unidentified ubiquitin ligase. (2) The Ubiquitin ligase transfers ubiquitin to Abl1 which interacts with the WDR domain of Nbeal2. (3) Abl1 is degraded via the proteasomal degradation pathway (These pictures were created by using Biorender at: https://www.biorender.com/). *Reference*: [1] Nagar B, Hantschel O, Young MA, Scheffzek K, Veach D, Bornmann W, et al. Structural basis for the autoinhibition of c‐Abl tyrosine kinase. Cell. 2003;112(6):859–71.

## Data Availability

The RNA sequencing data discussed in this publication were initially discussed by Wegner et al. [1], have been downloaded from NCBI's Gene Expression Omnibus through GEO Series accession number GSE225692 (sequencing data online available: https://www.ncbi.nlm.nih.gov/geo/query/acc.cgi?acc=GSE225692).
