# Supplementary figures and images for: Nbeal2 Inactivation Triggers Abl1 Stabilisation and Dysregulated Subcellular Localisation of the Multi‐Drug‐Resistant Protein MDR1 (ABCB1) in Mast Cells
Source: Immunology. 2025 Oct 19;177(2):355–69. doi: 10.1111/imm.70055 (PMC12779453; doi:10.1111/imm.70055)

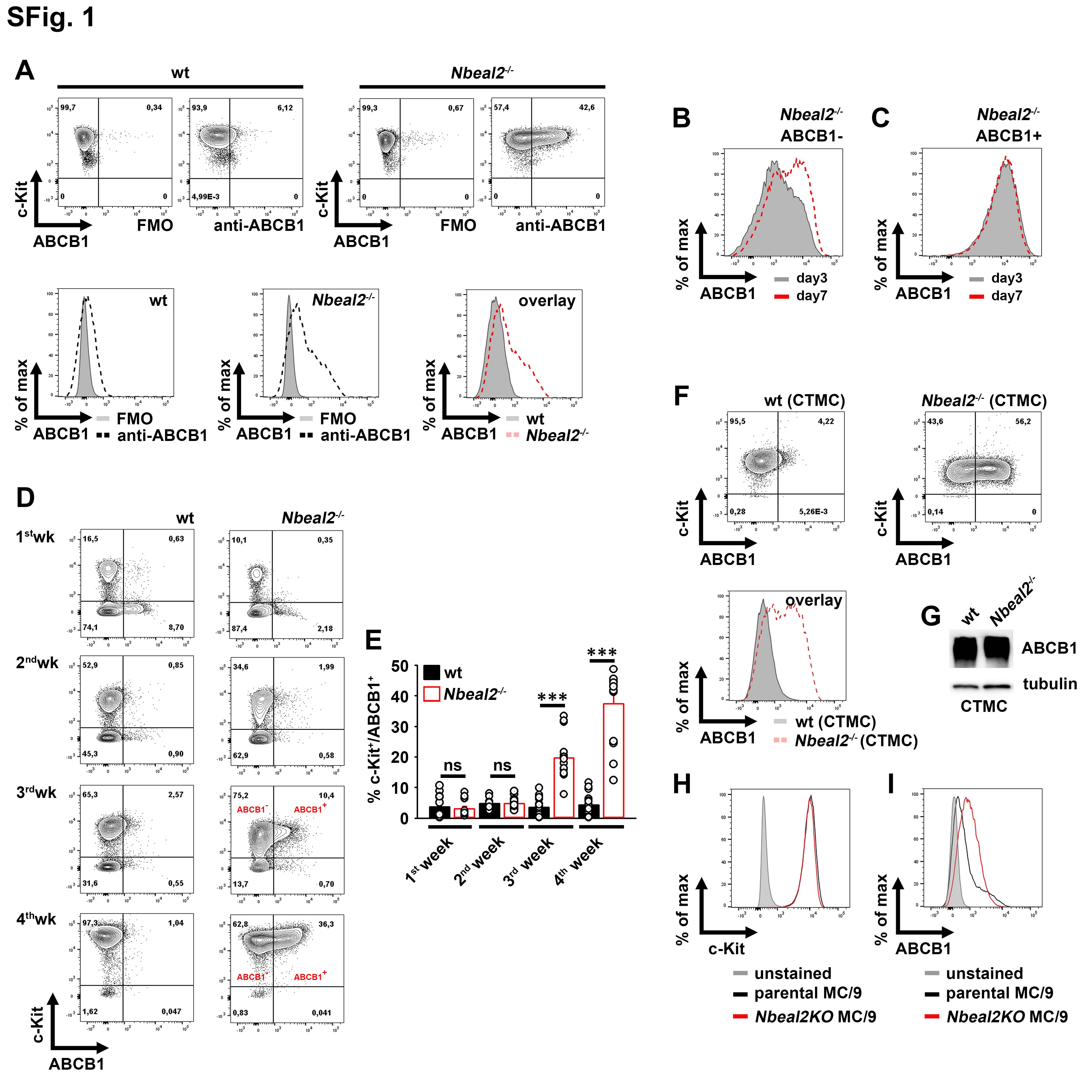

Supplement: Supplementary file 1 — Figure S1: Nbeal2 deficiency upregulates the surface expression of ABCB1. (A) Wt and Nbeal2 −/− BMMCs were either stained for c‐Kit alone (FMO for ABCB1 staining) or for c‐Kit and ABCB1. Cells were analysed by flow cytometry. FACS plots and histogram overlays are shown. (B, C) Nbeal2 −/− BMMCs were sorted for ABCB1. ABCB1− (B) and ABCB1+ (C) Nbeal2 −/− BMMCs were cultured for 3 or 7 days, were subsequently stained for ABCB1 and were analysed by flow cytometry. Overlays are shown. (D) Bone marrow cells from wt and Nbeal2 −/− mice were cultured in IL‐3 medium. After the indicated time points cells were stained for c‐Kit and ABCB1 and were analysed by flow cytometry. Representative FACS plots are shown. (E) Statistical analysis of (D) is shown (means ± SEMs of n = 15 biological replicates of wt and Nbeal2 −/− BMMCs). (F) Wt and Nbeal2 −/− CTMCs were stained for c‐Kit and ABCB1 and were analysed by flow cytometry. Flow cytometry plots and overlay of the ABCB1 expression of wt and Nbeal2 −/− CTMCs are shown. (G) Western blotting of lysates of wt and Nbeal2 −/− CTMCs are shown. (H, I) Parental and Nbeal2KO MC/9 cells were stained for c‐Kit and ABCB1 and were analysed by flow cytometry. Histograms of the ABCB1 expression are shown. [file IMM-177-355-s003.jpg]

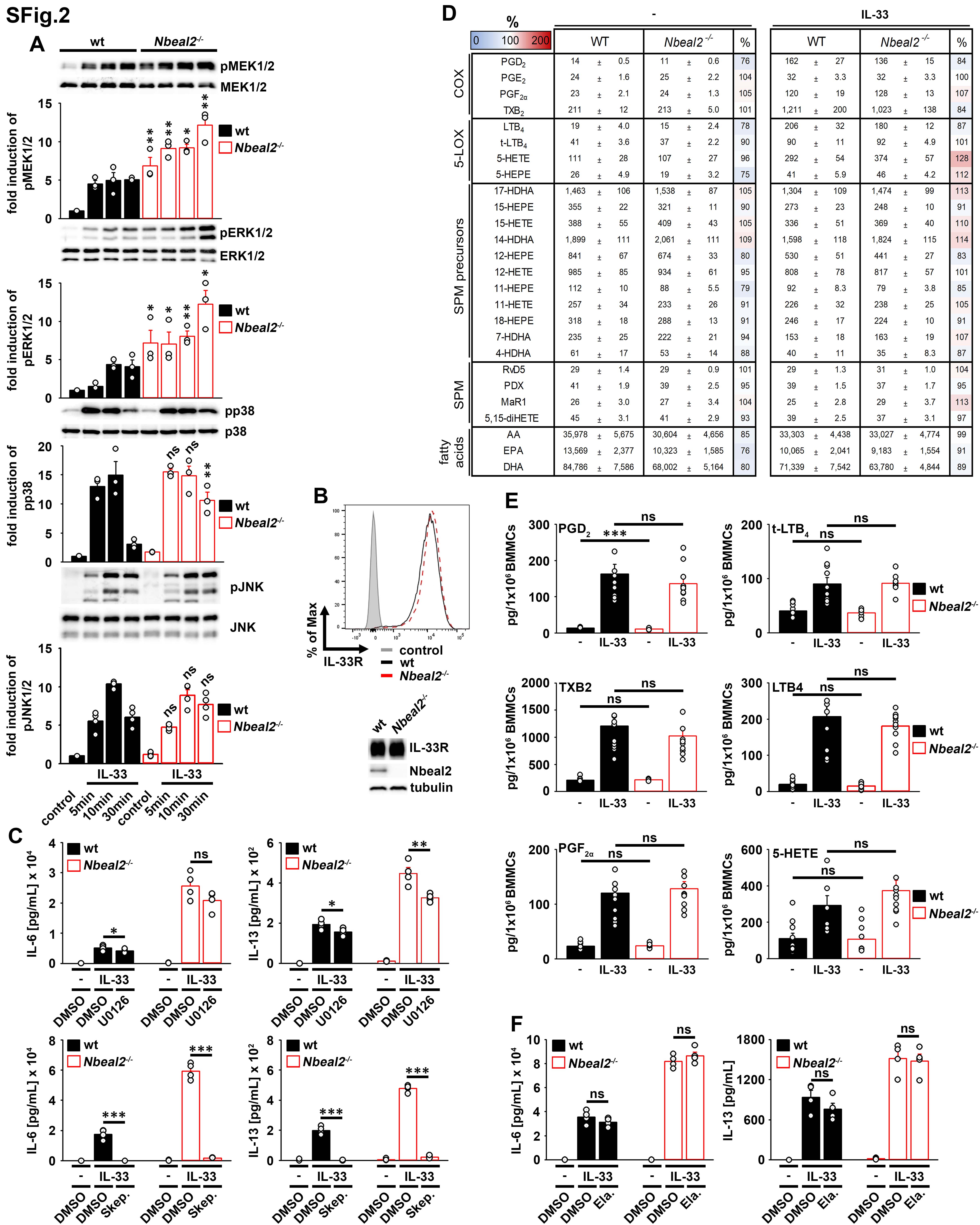

Supplement: Supplementary file 2 — Figure S2: Nbeal2 deficiency enhanced the IL‐33‐induced production of pro‐inflammatory cytokines via p38. (A) Wt and Nbeal2 −/− BMMCs were stimulated with IL‐33 (50 ng/mL) as indicated. Lysates were analysed by Western blotting. Statistical analysis of densitometric analysis are shown below each representative Western blots [means ± SEMs of n = 3 (for pJNK1/2, n = 4) biological replicates of wt and Nbeal2 −/− BMMCs; the statistics were performed between the respective time points in the wt and the Nbeal2 −/− groups]. (B, upper panel) Wt and Nbeal2 −/− BMMCs were stained for the IL‐33R and were analysed by flow cytometry. Shown is one representative flow cytometry experiment out of n = 3 biological replicates of wt and Nbeal2 −/− BMMCs. (B, lower panel) Wt and Nbeal2 −/− BMMCs were lysed and analysed by Western blotting. Shown are representative Western blots from n = 4 biological replicates of wt and Nbeal2 −/− BMMCs. (C) Wt and Nbeal2 −/− BMMCs were treated with vehicle (DMSO, as indicated) or either with U0126 (10 μM), or L‐Skepinone (5 μM) (Skep.). 30 min later, cells were stimulated with IL‐33 (50 ng/mL) for 24 h and supernatants were analysed by ELISA. Statistical analyses are shown (means ± SEMs of n = 4 biological replicates of wt and Nbeal2 −/− BMMCs). (D, E) Wt and Nbeal2 −/− BMMCs were stimulated with IL‐33 (50 ng/mL) for 24 h. Lipid mediators were extracted from the supernatant by solid‐phase extraction and analysed by UPLC‐MS/MS. Summarising overview is shown in the table (D) and the statistical analysis is shown in (E) (means ± SEMs of n = 10 biological replicates of Nbeal2 −/− BMMCs). (F) Wt and Nbeal2 −/− BMMCs were treated with vehicle (DMSO, as indicated) or with Elacridar (5 μM) (Ela.). 30 min later, cells were stimulated with IL‐33 (50 ng/mL) for 24 h. Supernatants were analysed by ELISA and statistical analyses are shown (means ± SEMs of n = 4 biological replicates of wt and Nbeal2 −/− BMMCs). [file IMM-177-355-s004.jpg]

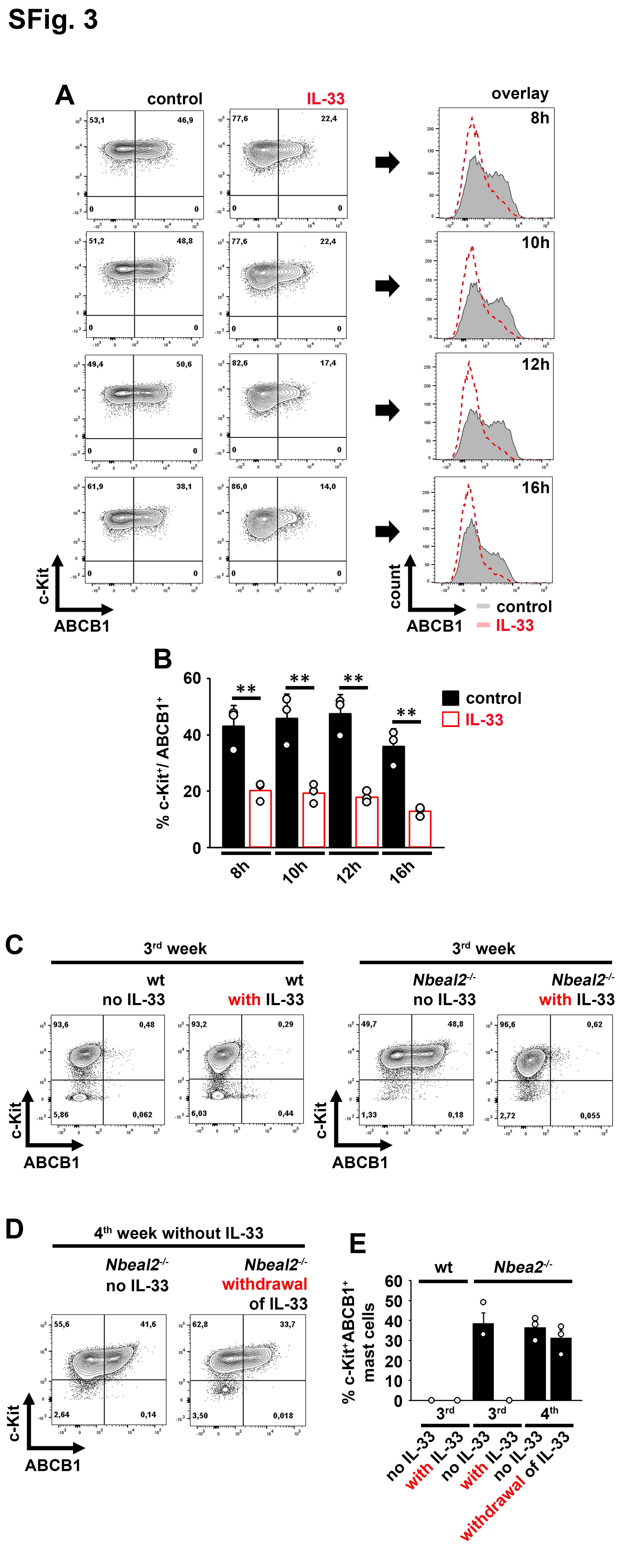

Supplement: Supplementary file 3 — Figure S3: IL‐33 reversibly regulates the ABCB1 expression on Nbeal2 −/− BMMCs. (A, B) Nbeal2 −/− BMMCs were stimulated with IL‐33 (50 ng/mL) for the indicated time periods. Subsequently, cells were stained for c‐Kit and ABCB1 and were analysed by flow cytometry. Representative plots and histogram overlays are shown. (B) Statistical analysis of (A) are shown in (means ± SEMs of n = 3 biological replicates of Nbeal2 −/− BMMCs). (C–E) Bone marrow cells from wt and Nbeal2 −/− mice were either cultured in IL‐3 medium (no IL‐33) or in medium containing IL‐3 and IL‐33 (with IL‐33). After 3 weeks, cells were stained for c‐Kit and ABCB1 and were analysed by flow cytometry. Representative plots are shown (C). (D) From the 3rd week on, IL‐33 was withdrawn from the cell culture and cells remained in basic culture conditions (with IL‐3 but without IL‐33) for another week. After the 4th week, cells were stained for c‐Kit and ABCB1 and were analysed by flow cytometry. Representative plots are shown. (E) Statistical analysis of (C, D) are shown (means ± SEMs of n = 3 biological replicates of wt and Nbeal2 −/− BMMCs). [file IMM-177-355-s002.jpg]

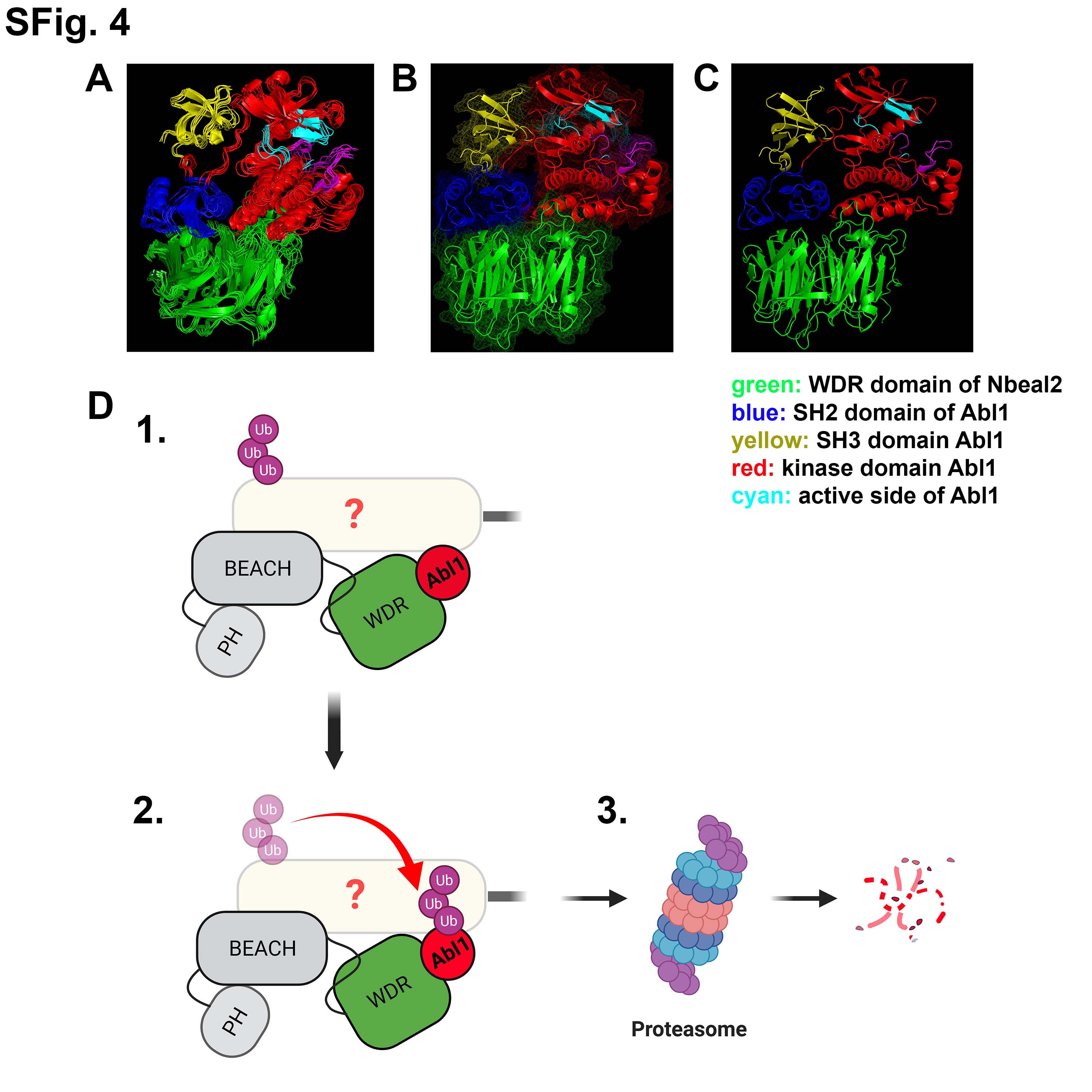

Supplement: Supplementary file 4 — Figure S4: The proposed model for the NBEAL2/ABL1 interaction and the proposed model for the Nbeal2‐mediated Abl1 degradation in MCs. (A) The interaction of the WDR domain of NBEAL2 (AA 2371–AA 2754) with ABL1 (AA 60–AA 500) was predicted by using Boltz‐1 (AlphaFold3) at: https://neurosnap.ai. Thereby, we obtained 5 possible interaction structures models with a high confidence each. These 5 structures models were merged by using the pymol software. The ABL1 structure obtained by Boltz‐1 (AlphaFold3) is similar to the structure obtained by x‐ray diffraction analysis of ABL1 [1]. (B, C) One of the 5 interaction structure models was randomly chosen and is shown in a mesh/ribbon (B) and ribbon (C) presentation. (D) (1) In MCs, we propose that Abl1 interacts with the WDR domain from Nbeal2. This Nbeal2/Abl1 complex interacts with a yet unidentified ubiquitin ligase. (2) The Ubiquitin ligase transfers ubiquitin to Abl1 which interacts with the WDR domain of Nbeal2. (3) Abl1 is degraded via the proteasomal degradation pathway (These pictures were created by using Biorender at: https://www.biorender.com/). Reference: [1] Nagar B, Hantschel O, Young MA, Scheffzek K, Veach D, Bornmann W, et al. Structural basis for the autoinhibition of c‐Abl tyrosine kinase. Cell. 2003;112(6):859–71. [file IMM-177-355-s001.jpg]
